# Effects of Social Attitude Change on Smoking Heritability

**DOI:** 10.1007/s10519-017-9871-1

**Published:** 2017-09-25

**Authors:** Laura Mezquita, Juan F. Sánchez-Romera, Manuel I. Ibáñez, José J. Morosoli, Lucía Colodro-Conde, Generós Ortet, Juan R. Ordoñana

**Affiliations:** 1 Department of Basic and Clinical Psychology and Psychobiology, Universitat Jaume I, Av. de Vicent Sos Baynat, s/n, 12071 Castelló, Spain; 2 Centre for Biomedical Research Network on Mental Health (CIBERSAM), Instituto de Salud Carlos III, Madrid, Spain; 3 Departament of Human Anatomy and Psychobiology, Universidad de Murcia, Murcia, Spain; 4 Murcia Institute for Biomedical Research, IMIB-Arrixaca, Murcia, Spain; 5 Department of Genetics and Computational Biology, QIMR Berghofer Medical Research Institute, Brisbane, Australia

**Keywords:** Smoking, Sex differences, Genetic factors, Gene-environment interactions, Twin study, Spain

## Abstract

Societal attitudes and norms to female smoking changed in Spain in the mid-twentieth century from a restrictive to a tolerant, and an even pro-smoking, posture, while social attitudes remained stable for males. We explored whether this difference in gender-related social norms influenced the heritability of two tobacco use measures: lifetime smoking and number of years smoking. We used a population-based sample of 2285 twins (mean age = 55.78; SD = 7.45; 58% females) whose adolescence began between the mid-1950s and the early 1980s. After modeling the effect of sex and year of birth on the variance components, we observed that the impact of the genetic and shared environmental factors varied differently by birth cohort between males and females. For females, shared environment explained a higher proportion of variance than the genetic factors in older cohorts. However, this situation was inverted in the younger female cohorts. In contrast, no birth cohort effect was observed for males, where the impact of the genetic and environmental factors remained constant throughout the study period. These results suggest that heritability is larger in a permissive social environment, whereas shared-environmental factors are more relevant in a society that is less tolerant to smoking.

## Introduction

Tobacco primarily influences health negatively by favoring heart, respiratory and cardiovascular disease, and lung cancer, among others (Maritz and Mutemwa 2012). Tobacco kills nearly 6 million people each year worldwide, and around 16% of deaths in Europe are attributed to it (WHO 2013). For these reasons, knowing which factors contribute to individual differences in tobacco use is essential to adopt prevention and treatment strategies.

Behavior genetics studies support the notion that genetic factors and shared environment are mainly responsible for individual differences in smoking initiation, while the influence of unique environment increases when smoking becomes a regular habit, and includes measures of the amount of tobacco smoked, regular use, dependence and persistence (Li et al. 2003). Hence the shared environment influence decreases if we compare smoking initiation to regular tobacco use, at least in males (Li et al. 2003). However, these estimations are not static. From a social epidemiological perspective, an individual’s location within a particular social structure is a fundamental determinant of vulnerability and exposure (Boardman et al. 2013). Consequently, it has been hypothesized that changes in the macro-environment (e.g., gender inequalities regarding attitudes toward smoking) may modify the heritability of tobacco outcomes, which suggests gene × environment interactions (G × E) (Short et al. 2013; Perry 2016).

Accordingly, different theories that explain G × E have been described. The *social control model* defends that social forces wash out the effect of genetic factors (Shanahan and Hofer 2005; Vink and Boomsma 2011; Boardman et al. 2013). That is, when there is social pressure to not smoke, the variability in the phenotype of genetically diverse individuals would narrow, then environmental factors would mostly explain individual differences in smoking. Along this line, there is evidence that the genetic influence on smoking is weaker in areas that pose relatively high taxes on cigarettes, and stricter controls on vending machines and cigarette advertising (Boardman 2009), in strong religious societies (Timberlake et al. 2006), and in societies that ban smoking in public places (Boardman et al. 2010). However, a recent report found no effect of social pressure to quit smoking on smoking heritability in young adult twins (Vink and Boomsma 2011). These authors suggested that the effects of social control on heritability would be specific of samples of regular smokers rather than on samples with a short smoking history. This idea has been supported by studies which have found that the genes associated with smoking initiation may differ from those associated with regular tobacco use (Broms and Silventoinen 2006; Hardie et al. 2006).

A second model is the *social trigger model*, which postulates that genetic factors differentiate between individuals in the presence of social pressure (Shanahan and Hofer 2005; Vink and Boomsma 2011; Boardman et al. 2013). Accordingly, some evidence suggests that daily smoking heritability is greater in students from high schools where the most popular students smoke the most (Boardman et al. 2008), when smoking emerged from a disreputable activity limited to marginal groups to one being accepted in more conventional middle-class groups (Boardman et al. 2010), and when smoking became more conventional among females (Kendler et al. 2000). In all these cases, pro-smoking norms act as a trigger for relative genetic influence.

That is, if the social environment makes smoking difficult for everyone, it inhibits the potential for genes to affect smoking (*social control model*), but if the social environment presents new choices, it facilitates the potential for genes to affect smoking (*social trigger model*) (Boardman et al. 2010). These two models attribute a causal influence of the social environment on limiting and exacerbating the salience of genetic influences.

A third model characterizes the environment across a full continuum, and is not necessarily causal. The *social push model* defends the idea that changes in social norms on smoking can affect the relevance of genetic influences by minimizing or maximizing “noise” with the potential to overwhelm and hide such influences (Boardman et al. 2010; Vink and Boomsma 2011). Namely, when smoking becomes a social phenomenon that pushes the whole population to smoke (regardless of genetic makeup), genetically vulnerable persons would be no more likely to begin smoking than genetically resilient persons simply because of the predominant social popularity of smoking. In contrast, if social influences discourage smoking, then genetic influences would increase in salience because quitting is physiologically harder for some people than it is for others (Boardman et al. 2010). In line with this, there is evidence that the genetic influence of smoking decreased between the mid-1930s and the mid-1940s in the United States, when tobacco became cheap and images of cultural icons smoking were published (Boardman et al. 2010). However, the genetic factors for quitting smoking became more important following restrictive legislation on smoking behaviors in the early and mid-1970s in the United States (Boardman et al. 2011). Recent molecular genetics reports have also found similar results and support this hypothesis (Domingue et al. 2016).

In the present research, we explored whether a change in the environment (i.e., change in the social attitude and norms towards female smoking) is related to a change in the heritability of two measures of tobacco use: *lifetime smoking* and *number of years smoking*. That is, if gender, understood as a concept that reflects differences in social roles between males and females, influences the heritability of two different smoking measures related to health (Short et al. 2013; Perry 2016).

Around the 1970s, a rapid social, political and economic transformation took place in Spain, which included the rapid evolution and improvement in women’s living conditions and opportunities. In this female empowerment scenario, inequalities between males and females decreased in many aspects (e.g., labor force participation, or increasingly equal access to Higher Education), which created opportunities for the tobacco industry to specifically target women using emancipation imagery by depicting smoking as a symbol of success and gender equality (Bilal et al. 2015). These changes were related to increased tobacco use among women.

**Fig. 1:**
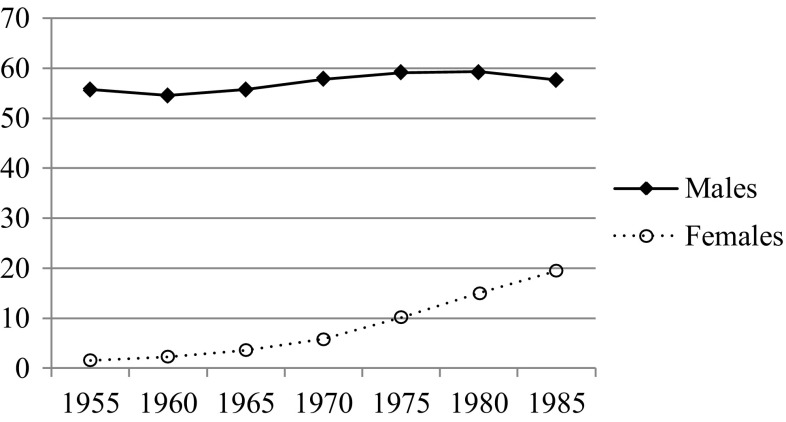
Current smoking prevalence (%) among ≥ 16-year-old Spanish males and females from 1955 to 1985 Adapted from Fernandez et al. (2003)

Before the early 1970s, smoking prevalence among females was very low in Spain (3.6% in 1965), but steadily increased after this period until the mid-1980s (19.5%). However in males, even with some peaks appearing in smoking prevalence, it became higher and stabler (between 55.7 and 57.6%) than in females over the same period (Fernández et al. 2003) (see Fig. 1). These researchers have shown that: (a) despite some delay, this pattern is similar to that reported in other developed countries like the US (Fernández et al. 2003); (b) the instauration of the highest tobacco prevalence in females was delayed by 20–30 years compared with males (Fernández et al. 2003); (c) the females born in a more gender equal context display smoking prevalence patterns that emulate those of males (Bilal et al. 2015).

The Murcia Twin Registry (MTR) is a population-based registry in Spain designed to analyze the relative contribution of genetic and environmental factors to the development of complex phenotypes, and focuses on health and health-related behaviors (Ordoñana et al. 2013). The twin pairs that form part of the MTR are assumed to be representative of the general population in its reference area (Ordoñana et al. 2017). They are particularly relevant for studying gene-environment interactions related to tobacco use patterns because they were born between 1940 and 1966. Thus the adolescence of a part of them began at a time when smoking was stigmatized among females, and it was when they first came into contact with smoking (the birth cohorts from 1940 to 1955), while others were socialized about smoking when a pro-smoking change in females occurred in Spain (the birth cohorts from 1956 to 1966). We specifically hypothesized that tobacco use heritability of females would increase according to changes in the macro-environment toward more permissive norms for female smoking, which indicates a G × E effect. Accordingly, we expected the heritability of tobacco outcomes among males to be stable over time. Sex differences in the sources of variance of smoking behavior were also explored.

## Methods

### Sample and procedure

The smoking data were collected by telephone interviews in 2013 as part of the third wave of data collection accomplished by the MTR (*N* = 1618, 55.1% females). They were 47 to 73 years old (mean age = 56.75, SD = 7.11). In order to increase sample size, the data about lifetime smoking from wave 1 (2007; *N* = 178, 100% female, mean age = 52.20, SD = 7.71) and wave 2 (2009–2011; *N* = 491, 52.34% female, mean age = 53.80, SD = 7.67) were also incorporated to the study when data from 2013 were not available (see the "Measures"). Thus the total sample was composed of 2285 twins (mean age = 55.76, SD = 7.45, 57.99% females). The number of twins, their zygosity and sex for each measured variable are represented in Table 1.

**Table 1:** Distribution of the participants (individuals) in the two study variables in the whole sample

	N	% Never smoked	% I smoke, or have smoked in the past		N	% 0 year smoking	% 1–20 year smoking	% > 20 year smoking
Lifetime smoking				Years smoking to 47 years old				
Males				Men				
MZ	285	28.07	71.93	MZ	223	31.39	15.69	52.91
DZss	354	29.66	70.34	DZss	259	33.20	18.15	48.65
DZos	321	24.61	75.39	DZos	235	28.93	11.49	59.57
Total	960	27.50	72.50	Total	717	31.24	15.20	53.56
Females				Women				
MZ	483	52.80	47.20	MZ	332	56.32	8.13	35.54
DZss	493	58.42	41.58	DZss	303	60.07	6.93	33.00
DZos	349	55.01	44.99	DZos	236	57.63	9.32	33.05
Total	1325	55.47	44.53	Total	871	57.98	8.04	33.98

Male–female difference for *lifetime smoking: χ* ^2^ = 214.55, *p* < .001; and for *years smoking: χ* ^2^ = 120.48, *p* < .01

*MZ* monozygotic, *DZ* dizygotic, *DZss* same sex dizygotic, *DZos* opposite-sex dizygotic

### Measures

Tobacco use was assessed by asking two questions. The first one was a lifetime smoking measure in which participants answered the question: “Do you smoke or have you ever smoked?”. Answers were coded as 0 “Never smoked”, and 1 “I smoked, but quitted” or “I still smoke”.

The number of total number of years smoking was collected with the question: “Could you tell us about the different periods of your life during which you smoked? For example, from year × to year Y”. As older participants were able to report a larger number of smoking years, the total number was calculated up to the age of 47, according to the youngest age of the participants assessed in 2013. Due to the non normal data distribution, the variable was recorded as ordinal with three levels: 0, 1 to 20 years, and more than 20 years.

### Statistical analysis

#### Preliminary analyses

First, a descriptive analysis of the two smoking variables was performed. Then the associations between sex and age, their interaction as predictors, and the two variables of interest were examined using generalized estimating equations (GEE) for the binary and ordinal data. Twin pairs cannot be assumed independent, so GEE was used to control for the clustering of twins within a pair. Descriptive analyses and a GEE procedure were performed with IBM SPSS Statistics 24 (IBM Corp. 2016).

#### Quantitative genetic modeling

Genetic analyses were conducted using the OpenMx package, v2.7.9 (Neale et al. 2016) for R v3.3.3 (R Core Team 2017). The classic twin design decomposes phenotypic correlations between traits into a combination of additive genetic (A), dominant genetic (D), shared environmental (C), and residual (E) factors (Rijsdijk and Sham 2002). We tested whether monozygotic (MZ) twin correlations were higher than those of dizygotic (DZ) twin pairs, which would suggest a genetic influence on the individual differences in this trait. It is not possible to estimate C and D simultaneously with twin data only because C and D are negatively confounded. The choice of modeling C or D depends on the pattern of the MZ and DZ correlations; C is estimated if the DZ twin correlation is more than half the MZ twin correlation, and D is estimated if the DZ twin correlation is less than half the MZ correlation (Neale et al. 2006). Therefore, we calculated the intra-pair polychoric twin correlations for each zygosity group, and either the ACE or ADE model was fitted. We also compared the former with other competing models: the CE, AE and E models.

Structural equation modeling was used to partition the variation in the two variables of smoking behavior into genetic and environmental sources. All the SEM models were fitted to the raw data by employing the full information maximum likelihood (FIML) method using OpenMx. The accuracy of the obtained parameters was assessed using likelihood-based 95% confidence intervals (LBCI) (Neale and Miller 1997).

Modeling included two analysis stages. First, a classic univariate twin model was performed to estimate the impact of genetic and environmental factors on *lifetime smoking* and *number of years smoking*. In this stage, models were fitted separately for males and females due to the different pattern of twin correlations observed by sex. Both variables were analyzed by a liability-threshold model (Rijsdijk and Sham 2002).

Second, in order to investigate G × E, we combined the general sex-limitation model (Neale and Maes 2004) with the Purcell approach for the binary and continuous moderators (Purcell 2002), and applied them to *lifetime* and *number of years smoking*. We assessed the qualitative sex differences in the contribution of common environmental factors by specifying a sex-specific C component for females. This is possible given the availability of the data from the dizygotic opposite-sex (DZos) twins. Higher DZss correlations than DZos correlations would suggest that different genes or shared environmental factors could influence the individual differences in this trait for each sex (Vink et al. 2012). Quantitative sex differences were modeled by specifying different sets of parameters (*a, c* or *d*, and *e*) for males and females.

**Fig. 2:**
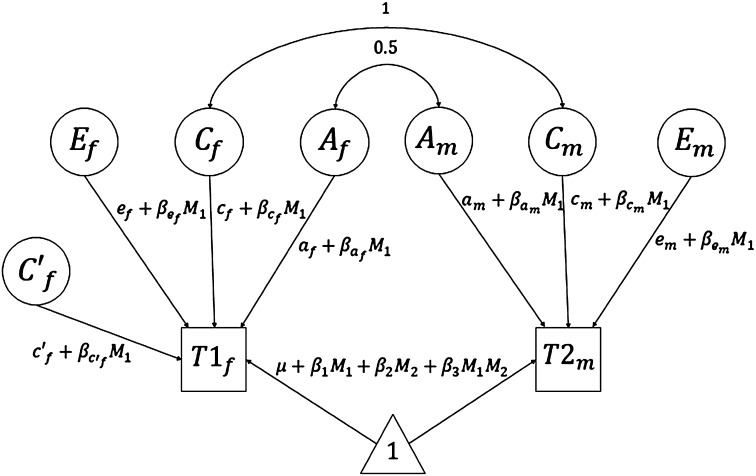
Univariate biometric moderation model with a female-specific shared-environmental component (C′_f_) and year of birth (M_1_) as moderators. In the model of means, M_1_ and M_2_ denote the covariates year of birth and sex, with their respective beta coefficients β_1_ and β_2_; and β_3_ for their interaction

The impact of the birth cohort on A, C (both common and female-specific factors) and E was analyzed by introducing year of birth as a moderator on each path (see Fig. 2).

In order to obtain the unstandardized estimates, we followed the method proposed by Medland et al. (2009) for the G × E analysis with categorical variables, and we constrained the variance of the dichotomous variable to be 1 at the mean of the moderator (the moderator was standardized), and the two thresholds of the ordinal variable to be 0 and 1, which allowed the total variance and mean to be free.

As the moderator of interest (year of birth) is shared by both members of each twin pair, the false-positive rate showed by van der Sluis et al. (2012) in some cases of this model type was no concern. In addition, the effects of age, sex, and their interaction, were regressed out from the raw scores by also following the FIML procedure in OpenMx.

To our intents and purposes, a series of nested models was performed. Differences in χ^2^ and AIC were calculated to estimate the significance of the differences in fit between models. In Model 1, the shared environmental (female-specific), component (${C^\prime}_{f}$) was estimated as a free parameter, along with an interaction coefficient (${\beta }_{{C^\prime }_{f}}$). In Model 2, the significance of the interaction between cohort and ${C^\prime }_{f}$ was tested by fixing ${\beta }_{{C^\prime }_{f}}$to 0. If no degradation in the model fit occurred, it would indicate that there was no evidence for changes in the presence of sex-specific C factors due to year of birth. In Model 3, the qualitative sex differences were tested by dropping the ${C^\prime }_{f}$ path. In Models 4, 5 and 6, the interaction between the birth cohort and the male, female and both sex groups set of parameters (${a}_{f,m}$, ${c}_{f,m}$, ${e}_{f,m}$) was respectively tested by fixing the interaction coefficients for each path to zero. Given our interest in the effect of the moderator on all three components, the interaction coefficients for each sex (${\beta }_{{A}_{f,m}}$, ${\beta }_{{C}_{f,m}}$, ${\beta }_{{E}_{f,m}}$) were dropped together at a time. Finally in Model 7, the quantitative sex differences were tested by equating the estimates of A, C and E, for males and females.

Since the goodness-of-fit of a model to the observed data is distributed as a Chi square (χ^2^), by testing the change in the Chi-square (Δχ^2^) against the change in degrees of freedom (Δdf), we can test whether dropping or equating specific model parameters significantly worsens the model’s fit. The best fitting model was chosen in each case by deducting the residual deviance of the compared models and by comparing Akaike’s information criterion (AIC).

In order to avoid incorrectly ruling out any variance component due to an insufficient sample size to detect its effect (Sullivan and Eaves 2002), the best fitting model retained all its parameters, and no AC, CE or E submodels were fitted in this second set of analyses.

## Results

### Descriptive analysis

**Fig. 3:**
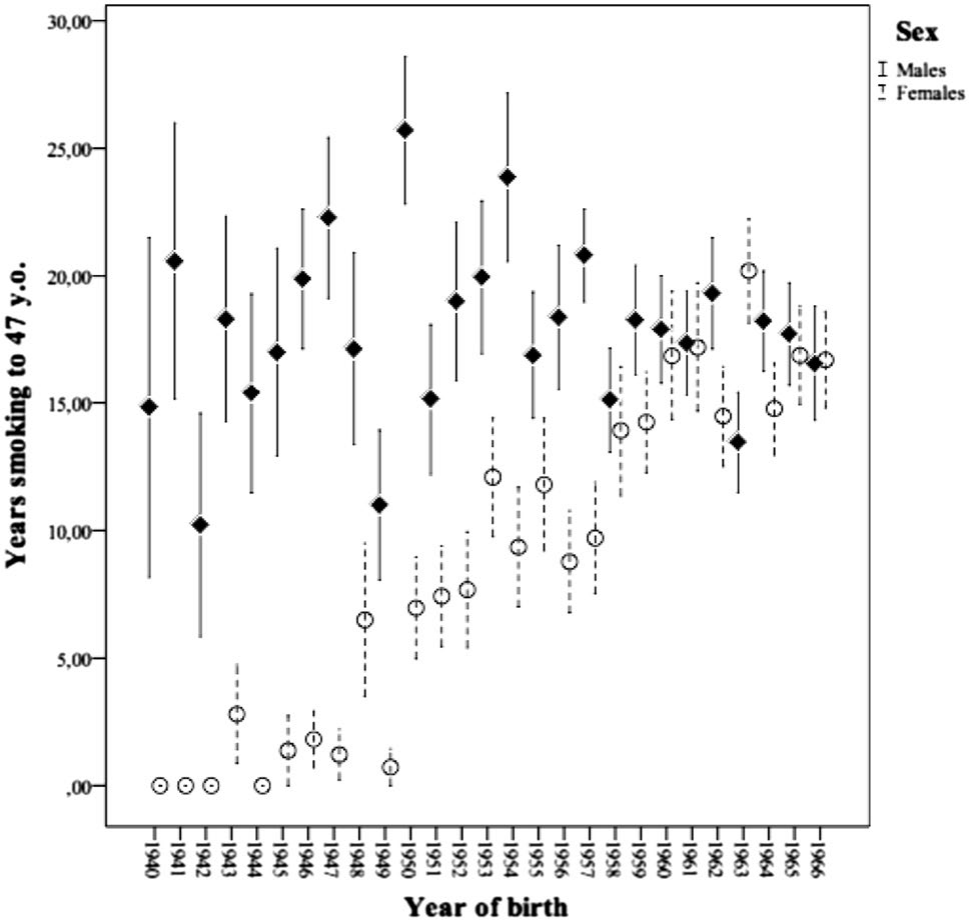
Error bar plot of mean *years of smoking to 47* years old with ±1SE by year of birth

We found that among the full sample of smokers, the majority (56.2%) started to smoke as adolescents (median = 17 years old, range: 7–56 years old), and that 83.7% smoked at 20 years old. The response frequencies of the two phenotypes in the whole sample, stratified by sex, are presented in Table 1. In addition, the graphical representation of the number of years smoking to 47 years old in the different years of birth and sex groups is presented in Fig. 3.

Regarding the GEE logistic regression analysis, a significant main effect of sex was found for *lifetime smoking* [OR 0.298, p < .001, 95% CI (0.242 0.367)] and for *number of years smoking* [OR 0.354, p < .001, 95% CI (0.283 0.443)], while no significant effect of age was found for either of the two variables [OR 2.416, p = .621, 95% CI (0.885 1.227), and OR 2.730, p = .998, 95% CI (0.845 1.178), respectively]. However, in the two variables, the interaction effect sex × age was significant [OR 2.416, p < .001, 95% CI (1.944 3.002), and OR 2.730, p < .001, 95% CI (2.143 3.478), respectively].

### Assumption testing

All the thresholds could be constrained to be equal within twin pairs and across zygosity without significantly worsening fit for *lifetime smoking* and the means and variances for *years smoking* to 47 years old. The thresholds could not be equated across sex for *lifetime smoking*, but this was possible for the means in *number of years smoking*.

### Twin correlations

Twin correlations are shown in Table 2. In males, the MZ twin correlations were consistently stronger and generally twice the DZ correlations, which suggests that additive genetic factors could explain individual differences in both the tobacco use measures. The twin correlations among females were higher than for males in the two variables for both MZ and DZ twins. Nonetheless, the fact that the DZ correlations were higher than half the MZ correlations also suggests that shared environmental influences partly explain the variance in the studied phenotypes. The comparison of the DZss and DZos correlations indicated that there could be qualitative sex differences in traits.

**Table 2:** Polychoric and intraclass correlations with a 95% confidence interval (95% CI) for the two study variables

	Lifetime smoking			Years smoking to 47 years old		
	N pairs	r	95% CI	N pairs	r	95% CI
MZ Males	157	.87	.72–.95	121	.82	.65–.92
DZss Males	193	.45	.21–.65	140	.39	.10–.61
MZ Females	251	.88	.79–.94	183	.91	.84–.96
DZss Females	266	.63	.46–.77	166	.73	.59–.83
DZos	390	.27	.06–.47	265	.25	.06–.44

*MZ* monozygotic, *DZ* dizygotic, *DZss* same sex dizygotic, *DZos* opposite-sex dizygotic

### Univariate twin analysis

For *lifetime smoking*, additive genetic influences and a common environment were both important in females (h^2^ = 49%, c^2^ = 39%), and neither of them could be dropped from the model without significantly worsening fit (*p* = .003 and *p* = .035, respectively). For males, a weaker influence of C was found (h^2^ = 61%, c^2^ = 11%), and there were no differences between an ACE and an AE model (*p* = .909).

For *number of years smoking to 47* years old, the best fitting model was an AE model. Estimated heritability was 88% for females and 79% for males, whereas the remaining variance could be explained by unique environmental or stochastic factors. All the estimates, with 95% confidence intervals, and model comparisons are presented in Table 3.

**Table 3:** Model-fitting results for the two study variables and proportions of variance explained by additive genetic (A), common environment (C) and residual variation (E) with 95% confidence intervals (95% CI)

	A [95% CI]	C [95% CI]	E [95% CI]	−2LL	*df*	AIC	∆X^2^	∆*df*	*p*
Males									
Lifetime smoking									
ACE	.61 [.21 .81]	.11 [.00 .44]	.28 [.19 .42]	698.961	634	−569.038			
**AE**	.**87 [.73 .95]**	**–**	.**13 [.05 .26]**	**698.975**	**635**	−**571.025**	.**013**	**1**	.**909**
CE	–	.66 [.52 .77]	.34 [.22 .48]	71.263	635	−559.737	11.302	1	< .001
E	–	–	1	765.792	636	−506.208	66.830	2	< .0001
Years smoking to 47 years old									
ACE	.79 [.28 .89]	.00 [.00 .44]	.21 [.11 .36]	867.165	462	−56.835			
**AE**	.**79 [.64 .89]**	**–**	.**21 [.11 .36]**	**867.165**	**463**	−**58.835**	< .**0001**	**1**	**1**
CE	–	.60 [.45 .73]	.40 [.27 .55]	876.184	463	−49.816	9.019	1	.003
E	–	–	1	919.035	464	−8.965	51.870	2	< .0001
Females									
Lifetime smoking									
**ACE**	.**49 [.17 .87]**	.**39 [.03 .67]**	.**12 [.06 .21]**	**1025.120**	**971**	−**916.880**			
AE	.89 [.81 .95]	–	.11 [.05 .19]	1029.545	972	−914.455	4.425	1	.035
CE	–	.77 [.68 .85]	.23 [.15 .32]	1033.910	972	−91.090	8.790	1	.003
E	–	–	1	1162.842	973	−783.158	137.722	2	< .0001
Years smoking to 47 years old									
ACE	.58 [.16 .93]	.28 [.00 .65]	.13 [.06 .26]	826.134	616	−405.866			
**AE**	.**88 [.77 .94]**	**–**	.**12 [.06 .23]**	**827.587**	**617**	−**406.413**	**1.453**	**1**	.**228**
CE	–	.74 [.62 .84]	.26 [.16 .38]	833.474	617	−40.526	7.340	1	.007
E	–	–	1	904.960	618	−331.040	78.826	2	< .0001

Bold values indicates the best fitting model

*AIC* akaike information criterion, *df* degrees of freedom, *−2LL* twice negative log-likelihood, *∆X* ^*2*^ difference in X^2^ compared to the ACE model, *∆df* difference in degrees of freedom compared to the ACE model

### G × E and sex-limitation models

Table 4 shows the comparison of the different models that we specified for testing the significance of effect of sex and year of birth on the variance components. The same conclusion was reached for the two variables: we found no significant differences in fit when comparing the model with no moderation on the female-specific, shared environmental component (Model 2) to the general sex-limitation model. Similarly, the model with no sex-specific component at all (Model 3) did not significantly differ from Model 2. All together, these findings suggest no qualitative sex differences for any study variable.

**Table 4:** Comparison of the general and restricted sex-limited ACE models for the two study variables

Model	Comparison	Lifetime smoking						Years smoking to 47 years old					
		−2LL	AIC	*df*	χ^2^	∆*df*	*p*	−2LL	AIC	*df*	χ^2^	∆*df*	*p*
Model 1		249.039	−2063.961	2277				2463.505	−597.495	1530			
Model 2	Model 1	249.069	−2065.931	2278	.031	1	.861	2464.303	−597.697	1531	.798	2	.372
Model 3	Model 2	249.069	−2067.931	2279	< .001	1	1	2453.246	−61.754	1532	−11.057	3	1
**Model 4**	**Model 3**	**2496.364**	−**2067.636**	**2282**	**6.294**	**3**	.**098**	**2443.676**	−**626.324**	**1535**	−**9.570**	**3**	**1**
Model 5	Model 3	2503.091	−206.909	2282	13.022	3	.005	2463.402	−606.580	1535	1.156	3	.017
Model 6	Model 4	2509.549	−206.451	2285	13.185	3	.004	2454.197	−621.197	1538	1.521	3	.015
Model 7	Model 6	2516.701	−2059.299	2288	7.152	3	.067	2477.741	−604.259	1541	23.544	3	< 0.001

Bold values indicate best fitting model

Model 1: general sex-limitation ACE model with ${C^\prime }_{f}$ and ${\beta }_{C^\prime f}$ freely estimated. Model 2: qualitative sex differences without moderation in the sex-specific component $\left({\beta }_{C^\prime f}=0\right)$. Model 3: no qualitative sex differences $\left({C^\prime }_{f}=0\right)$. Model 4: fixing interaction coefficients for males to be zero. Model 5: fixing interaction coefficients for females to be zero. Model 6: fixing interaction coefficients for males and females to be zero. Model 7: equating ACE for males and females

Constraining the estimates for the moderation of year of birth on the genetic, common and unique environmental factors for males (Model 4) did not deteriorate the model’s fit in either variable. This implies that the estimates did not significantly differ for that group over time. However, this was not the case for females as fixing the moderation coefficients to zero significantly worsened fit. The unstandardized estimates for Model 4 are presented in Table 5.

**Table 5:** Unstandardized genetic, environmental and beta path estimates and confidence intervals for the best fitting restricted sex-limitation model

	Lifetime smoking			Years smoking to 47 years old		
	Intercept					
	$a$	$c$	$e$	$a$	$c$	$e$
	$\left(95\%\;{\text{CI}}\right)$	$\left(95\%\;{\text{CI}}\right)$	$\left(95\%\; {\text{CI}}\right)$	$\left(95\%\; {\text{CI}}\right)$	$\left(95\%\;{\text{CI}}\right)$	$\left(95\%\; {\text{CI}}\right)$
Males	0.832 (0.554, 0.953)	−0.407 (−0.696, −0.019)	0.375 (0.239, 0.536)	1.937 (0.833, 2.716)	−1.597 (−2.387, −0.807)	1.387 (0.990, 1.926)
Females	0.871 (0.654, 0.931)	0.349 (−0.126, 0.658)	0.345 (0.245, 0.461)	−3.647 (−5.806, −1.476)	−3.988 (−6.040, −2.225)	−2.301 (−3.476, −1.535)

	Moderation coefficient					
	${\beta }_{a}$ $\left(95\%\;{\text{CI}}\right)$	${\beta }_{c}$ $\left(95\%\; {\text{CI}}\right)$	${\beta }_{e}$ $\left(95\%\;{\text{CI}}\right)$	${\beta }_{a}$ $\left(95\%\; {\text{CI}}\right)$	${\beta }_{c}$ $\left(95\%\; {\text{CI}}\right)$	${\beta }_{e}$ $\left(95\%\; {\text{CI}}\right)$
Males	−	−	−	−	−	−
Females	0.365 (0.070, 0.618)	0.493 (0.228, 0.702)	0.050 (−0.08, 8 0.169)	−2.171 (−3.954, −0.643)	−0.816 (−2.326, 0.498)	0.108 (−0.738, 0.950)

**Fig. 4:**
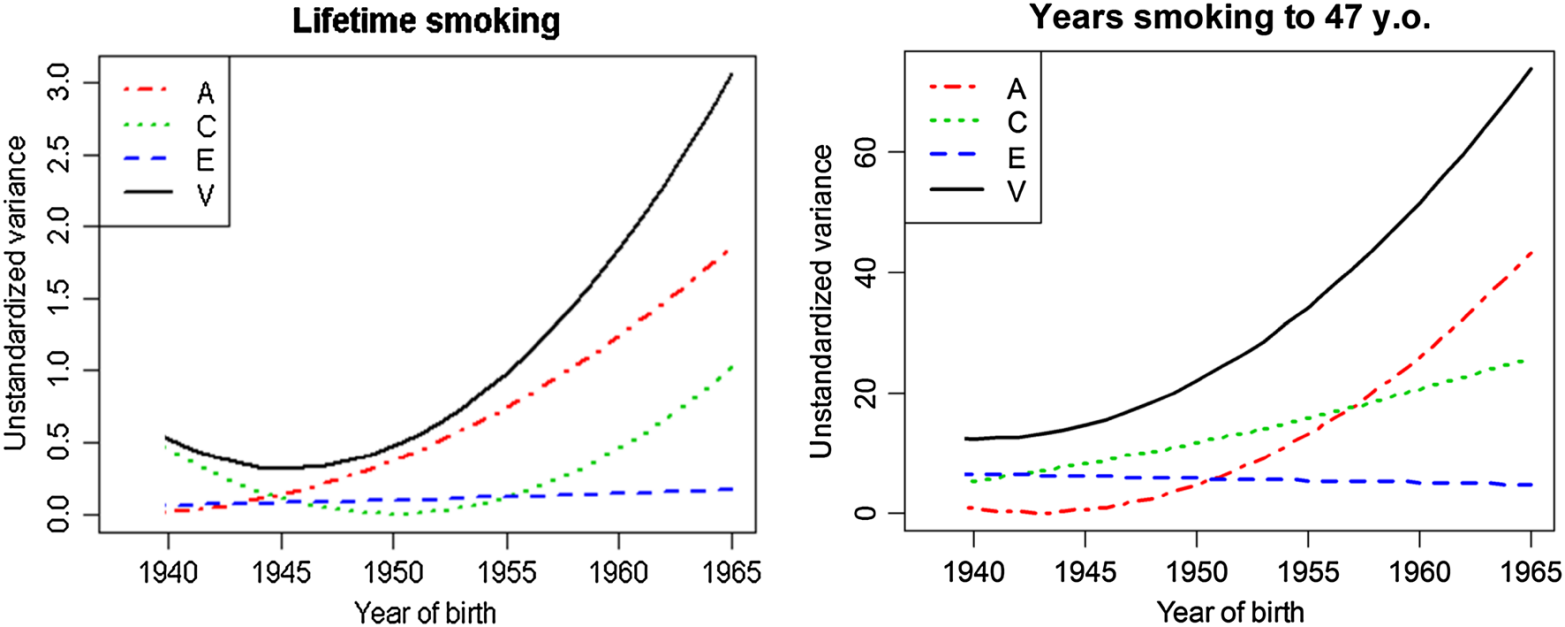
Effect of year of birth on the unstandardized variance components for both variables for females. Lines represent changes in the raw variance of components across years of birth

Therefore, the model that best fitted the data (Model 4) was that which included only the moderation effect of year of birth on the variance components for females, but implied a constant impact of A, C and E for males over time. The standardized estimates for males were h^2^ = 69.3%, c^2^ = 16.6% and e^2^ = 14.1% for *lifetime smoking*, and h^2^ = 45.6%, c^2^ = 31% and e^2^ = 23.4% for *years smoking to 47* years old. For females the change in the unstandardized variance components for both variables is shown in Fig. 4. The standardized estimates for the initial and final ends of the study period signal the observed variation: *lifetime smoking* (h^2^ = 0.1–60.8%; c^2^ = 92.5–33.6%; e^2^ = 7.4–5.6%); *years smoking to 47* years old (h^2^ = 15.9–58.6%; c^2^ = 33.5–35.1%; e^2^ = 50.6–6.3%).

## Discussion

This study explored smoking patterns and the influence of genetic and environmental factors in a sample of males and females who were socialized about smoking during the period that went from the mid-1950s to the early 1980s, when an attitudinal change toward increasing tolerance of female smoking took place in Spain. Such an attitude, however, remained mostly unchanged in males for the same years. It was hypothesized that the gender inequalities which took place during this period could modify not only the prevalence of the initiation and maintenance of smoking, but also the heritability of tobacco measures among females, which would indicate G × E. Any qualitative and quantitative sex differences in the sources of variance that could explain these phenotypes were also explored.

The distribution of different tobacco use patterns in the twin MTR sample, which is similar to the smoking prevalence reported for the Spanish population in previous studies (Fig. 1) (Fernández et al. 2003), indicated that social permissiveness at the time of first experiences with smoking had long-lasting effects on tobacco use throughout life. That is, the females who grew up in an environment in which female smoking was not popular displayed a reduced smoking pattern compared to those whose first experiences with tobacco took place in a more open society about female smoking. In line with this, stability in the male smoking prevalence in the Spanish society (Fernández et al. 2003) corresponded with the more stable tobacco use patterns found among our male cohorts.

Previous studies have indicated that Spanish males obtained higher scores in different tobacco use measures than females (National Plan of Drugs 2015). However, the differences we found were much more marked if subjects lived when adolescents in a society that had a less tolerant attitude to female smoking than for those who grew up in a society that actually considered female smoking an equality claim (see Fig. 3). By considering these results together with previous epidemiological studies, this pattern suggests that when social gender inequality diminishes (i.e., women’s rights movement, a less traditional family concept began, etc., around the 1970s), sex differences in smoking also decreased (Bilal et al. 2015).

When we estimated the relative contribution of the genetic and environmental factors to explain the individual differences in the two phenotypes, genetic factors and unique environment explained the individual differences found in the two phenotypes for both males and females, while shared environment also explained part of variance for lifetime smoking in females. The magnitudes of the variance component estimations that we found are similar to those reported in a previous meta-analysis about different tobacco use measures (Li et al. 2003).

When the moderation effect of year of birth and sex was modeled, differences in the relative contribution of the genetic and environmental factors among females, depending of their year of birth, were found and suggested G × E. These differences were statistically significant when formally tested (Model 4). These results suggest that environmental factors were likely to be mainly responsible for the individual differences in tobacco use in older females, while genetic factors were mainly responsible in the females born after the 1960s.

Our results contribute to the literature, which supports the notion that when stigmatization about smoking occurs, environmental factors explain individual differences in smoking among females (*social control model*). However, when social pressure to not smoke is relaxed over time, the pro-smoking attitude serves as a *trigger* for genetic influences (Kendler et al. 2000; Boardman et al. 2010). This shift reflects the change that took place in Spain during the studied decades. Spain went from a society that understood smoking as a properly adult male behavior and, consequently, banned female smoking, to become a social milieu where female smoking was progressively presented as a symbol of success, modernity and gender equality. In the first case, only the females on the extremes (very high or very low) of the distribution for such environmental factors, such as socio-economic status or educational attainment, smoked. Only when female smoking became progressively acceptable, or even desirable, and prevalence increased did genetic influence turn out to be salient.

Finally, in order to detect qualitative sex differences, and to explore if a similar mechanism accounted for the variation noted in the traits for males and females, comparisons of similarity between opposite-sex twin pairs with dizygotic same-sex twin pairs were made (Eaves et al. 1978). Although the twin correlations suggested qualitative sex differences in both phenotypes, these differences were not significant when formally tested in the structural equation models (Models 2 and 3). This result is similar to those obtained in a previous meta-analysis about the prevalence of current and lifetime smoking and nicotine dependence in adolescents and adults (Vink et al. 2012). By taking both studies together, it seems that the same genetic and shared environmental factors operate in male and female smoking, although their magnitude may vary.

The present research work has its limitations that must be taken into account. Firstly, in order to increase the sample size, non smokers were included in the analysis by considering smoking behavior as a continuum. This has been usually done in previous studies about tobacco use in adults (i.e., Carmelli et al. 1990; Kendler et al. 2000; see; Li et al. 2003), and helps to compare studies. Secondly, a larger sample size would have provided tighter confidence intervals for the moderation coefficients, and would have been more sensitive for detecting different G × Es. Finally, the participants were asked about smoking habits throughout their lives. Thus their answers could not be free of any recall bias.

In short, we studied changes in the influence of genetic and environmental factors on two tobacco use measures in relation to the social changes that occurred in Spain in the mid-twentieth century. Both sample characteristics and social contexts in which the participants were socialized about smoking conferred a valuable framework to study gene-environment interactions. Despite some limitations, the results of the studied phenotypes met our expectancies. When female smoking was restrained, individual differences in tobacco use among females were explained mainly by the environment (older birth cohorts). However, when smoking was freely accepted among females (younger birth cohorts), individual differences were explained mainly by genetic factors. These results suggest that changes in social attitudes toward smoking affect smoking heritability.
